# Line-dependent differences in locomotor performance: a comparative climbing analysis in wild-type and classical mutants of *Drosophila melanogaster*

**DOI:** 10.1080/19336934.2026.2760604

**Published:** 2026-11-09

**Authors:** Mehmet Fidan

**Affiliations:** Science and Art Center, The Ministry of National Education, Çorum, Türkiye

**Keywords:** *Drosophila melanogaster*, climbing assay, climbing speed, motor behaviour, genetic background, neuromuscular function, negative geotaxis

## Abstract

Negative geotaxis climbing assays are widely used to assess neuromuscular integrity in Drosophila melanogaster, but standardized comparisons across multiple classical mutants remain limited. We compared wild-type Oregon-R flies with four mutant lines (w1118, e1, vg1, and Ser1) at 5 cm and 17.5 cm thresholds, using 2,000 flies (5 lines × 2 sexes × 20 tubes × 10 flies). Climbing height and time-to-threshold were scored from video, and climbing speed (cm/s) was calculated. Tube-level means were analysed using Kruskal-Wallis with Bonferroni-corrected Dunn’s tests for line, and the Scheirer-Ray-Hare test for line × sex effects. Performance differed significantly among lines for all metrics (H ≈ 187–191, df = 4, all p < 0.001, ε2 > 0.93). Oregon-R performed best (16.25 ± 0.06 cm; long-distance speed 0.926 ± 0.011 cm/s), whereas vg1 flies were severely impaired (3.40 ± 0.07 cm; 0.029 ± 0.001 cm/s). The hierarchy Oregon-R > w1118 > e1 > Ser1 > vg1 was preserved across all metrics, in both sexes analysed as independent cohorts, in 2,000 split-half partitions and in 5,000 bootstrap resamples. Neither sex nor the line × sex interaction was significant. Because the lines were not backcrossed to a common background, the hierarchy reflects line-level differences that cannot be attributed exclusively to the focal mutations. These results support classical mutant lines as reproducible models for movement-related phenotypes.

## Introduction

The locomotor function of *Drosophila melanogaster* Meigen, 1830 has been long established as a sensitive and straightforward readout of potential defects in neuromuscular coordination and substrate metabolism. Among the behavioural tools available for assessing motor function, the negative geotaxis climbing assay is one of the most common and reproducible methods used to test motor function [1,2]. In this assay, flies that have been tapped to the bottom of a vial climb upward against gravity. Although the response itself is executed by the central nervous system and the leg musculature, performance in the assay is an integrated phenotype that also depends on metabolic state, diet, age and genetic background [1,3–5]. Revisions of this technique during the past 20 years have refined its precision, enabling subtle locomotion defects to be detected in mutants and disease models. Madabattula et al. [6] described a sensitized version of the climbing test that increased the vertical threshold to 17.5 cm and showed that this modification improved the sensitivity of the assay to early or mild neuromuscular deficits in neurodegenerative *Drosophila* models.

Motor performance in *Drosophila* flies depends on age and environmental challenge, but also on genetic background and body morphology. Specific mutations modifying colour, cuticle properties, or wing morphology might indirectly affect climbing ability via a change in energy budgeting, locomotor performance, or neurotransmission [3,7,8]. To give an example, mutations in the *white* gene, commonly characterized by a morphological loss of eye pigmentation, lead to interferences with dopaminergic and serotonergic signalling neurochemical systems that are fundamental for both visual and motor functions [9]. Similarly, *ebony* mutations inactivate glial metabolism of biogenic amines and induce reduced concentrations of dopamine and serotonin with concomitant behavioural changes, including decreased activity and abnormal pacing [10].

Morphological mutations in genes such as *vestigial* and *Serrate* affect wing and thoracic development by preventing the proper functioning of Notch-Wingless signalling and muscle attachment sites, which in turn leads to flight musculature weakening and deficient coordination [11–13]. While poor climbing may be easy to unravel in these cases, existing literature suggests that the flies’ baseline locomotor ability can be affected by a number of factors. However, these differences are rarely tested directly among classical morphological and neurotransmitter-related mutants under standard conditions for all lines used.

The *white*, *ebony*, *vestigial*, and *Serrate* mutations represent independent genetic axes of transformation: the first two implicate neurotransmitter metabolism and pigment physiology, while the last two pertain to developmental patterning and muscular attachment. Every such mutation opens a view to a restricted range of physiology: it is the neurochemical signal, the stress response, or morphogenetic coordination [9–11]. By comparing these mutant lines to the wild-type Oregon-R strain under a single protocol, it is feasible to quantify how much baseline motor performance varies among the classical lines that are routinely used as tools and models, and to establish a reference against which future background-controlled studies can be compared.

In light of these previous reports, changes in morphology, pigmentation, and/or neurotransmitter metabolism could individually be harboured by the respective mutant lines to compromise *Drosophila* motor fitness. Nevertheless, the respective roles of these different genetic backgrounds in baseline locomotor ability are not well characterized. By comparing mutants that have different developmental and neurochemical phenotypes, a comparative approach can shed light on how particular gene functions are reflected in quantifiable behavioural expressions [14–16].

To our knowledge, no prior study has directly compared these four classical mutant lines (*w*^1118^, *e*^1^, *vg*^1^, and *Ser*^1^) against a common wild-type reference within a single standardized design spanning two climbing-distance thresholds and both rate- and threshold-based outcome metrics; most published comparisons address these mutants individually or in smaller subsets. The two thresholds used here were selected to capture different levels of motor demand: a shorter 5 cm threshold, comparable to the moderate distances used in conventional negative geotaxis assays for detecting gross locomotor deficits [1], and a longer 17.5 cm threshold following the sensitized protocol of Madabattula et al. [6], which increases sensitivity to milder or early-stage neuromuscular impairment. Using both together allows the assay to capture deficits across this range of severity within the same set of flies.

Thus, this work aimed to investigate and compare the climbing performance of wild-type Oregon-R flies with four classical mutant lines representing distinct neurochemical and morphological deficits, *w*^1118^, *e*^1^, *vg*^1^, and *Ser*^1^, at short (5 cm) and long (17.5 cm) vertical thresholds. Gene and allele symbols follow FlyBase nomenclature [17]. We predicted that lines carrying mutations affecting neurotransmission (*w*^1118^, *e*^1^) or morphological development (*vg*^1^, *Ser*^1^) would show impaired climbing relative to wild-type flies, with the degree of impairment corresponding to the physiological system affected. Through quantitative climbing assays monitoring the interplay between line identity, morphology, and behaviour, this study aims to offer an integrative view on how structural and neurochemical disarrangements impact the walking capacity of *D. melanogaster*.

## Materials & methods

### Fly strains and rearing conditions

Five *D. melanogaster* lines were used in this study: the wild-type Oregon-R strain and four classical mutant lines carrying the alleles *w*^1118^ (FBal0018186; *white*), *e*^1^ (FBal0003278; *ebony*), *vg*^1^ (FBal0017868; *vestigial*), and *Ser*^1^ (FBal0015427; *Serrate*). FlyBase identifiers are provided for cross-referencing, and gene and allele symbols follow FlyBase nomenclature [17]. All lines were maintained as independent stocks in the FIDANS FLY LAB collection (Çorum, Türkiye), as in our previous work [18]; none of the mutant lines had been backcrossed to Oregon-R or to any other common background prior to this study, so each line carries its own historical genetic background in addition to the focal allele (see Discussion). All stocks were kept at 25 ± 1°C, under 60–70% relative humidity and a 12:12 h light-dark photoperiod, following standard maintenance protocols [1,2].

### Diet

All stocks and experimental flies were reared on the same cornmeal-sucrose-yeast-agar medium used in our previous work [18]. Per batch, 60 g sucrose and 9 g agar were dissolved in 440 mL water and brought to the boil. Separately, 50 g cornmeal and 19 g brewer’s yeast were suspended in 125 mL water, added to the boiling sucrose-agar solution, and the mixture was cooked for a further 10 min with continuous stirring (565 mL water in total; per-litre equivalents: 106 g sucrose, 16 g agar, 88 g cornmeal, 34 g brewer’s yeast). After cooling to approximately 60°C, 3 mL propionic acid (approximately 5 mL L^−1^) was added as a preservative. The medium was dispensed at 10 mL per vial, allowed to set, and stored at 4°C for no longer than 7 days. Medium was prepared weekly; all five lines and both sexes were reared on medium from the same batches, and test flies were kept on this medium from eclosion until testing at day 5 at a density of 20 flies per vial. Because dietary composition is known to alter negative geotaxis performance and other life-history traits in *Drosophila* [4,5], the use of a single, fully specified diet for all lines was an essential element of the standardized design.

### Experimental animals

Newly emerged adults were collected within 24 hours under light CO_2_ anaesthesia and separated by sex. Females were maintained as virgins in same-sex groups to prevent mating prior to testing, as mating status has been shown to influence locomotor performance in *Drosophila* females [3,19]. All experiments were conducted using five-day-old adults, which represent a stable physiological age for assessing motor performance [6]. For each of the 5 lines and 2 sexes (10 line-sex combinations), 20 independent replicate tubes were prepared, each containing 10 individuals (200 flies per line-sex combination; 2,000 total individuals; 400 individuals and 40 replicate tubes per line). Each replicate tube served as the independent experimental unit for statistical analysis.

### Climbing apparatus and experimental setup

Climbing performance was assessed using the negative geotaxis assay, a well-established method for quantifying locomotor ability in *Drosophila* [1]. A schematic of the apparatus and of the assay sequence is given in Figure 3. The test apparatus consisted of transparent glass tubes measuring 25 cm in height and 2.5 cm in internal diameter, with a rounded bottom and closed at the top with a foam plug, vertically mounted on a custom rack that allowed simultaneous evaluation of several replicate tubes. Two target heights were marked on each tube: 5 cm (short-distance threshold) and 17.5 cm (long-distance threshold), corresponding to moderate and high motor challenges, respectively [6,20]. The rack was placed in front of a white background panel to maximize contrast, and a centimetre ruler fixed to the panel, with the two thresholds marked, allowed vertical positions to be read directly from the recordings.

Flies were transferred gently into the tubes without anaesthesia in groups of 10 individuals per tube and allowed to acclimate for 10 minutes before testing. All 10 flies in a tube were tested simultaneously. At the start of each assay the tubes were tapped manually three times in rapid succession to bring all flies to the bottom; the observation window was timed from the last tap. For the short-distance assay, the maximum observation window was 10 s; for the long-distance assay, it was 120 s. Both assays were performed within the same session for each replicate tube, with the short-distance assay conducted first, followed immediately by the long-distance assay, so that both measures were obtained from the same group of flies under identical environmental conditions. Room temperature and relative humidity were recorded once per session and assigned to all 10 individuals tested in that session.

### Video acquisition and frame-based scoring

Each session was recorded with a DJI Osmo Pocket 2 camera (1920 × 1080 pixels, 60 frames s^−1^; temporal resolution 0.017 s) mounted on a tripod and positioned perpendicular to the rack, so that all replicate tubes tested in that session were within the same field of view (Figure 3A). Recordings were scored offline by a single observer by manually stepping through the video frame by frame in a standard video player; no automated tracking or image-analysis software was used. For each tube, the 10 flies were scored as 10 separate events: the time at which each fly first crossed the threshold line was read from the frame timestamp, giving 10 separate latency values per tube. Flies that had not crossed the threshold by the end of the observation window were assigned the window length as their time value, and their height was read from the final frame of the window against the ruler and the tube markings, to the nearest 0.1 cm. Because the four mutant lines carry externally visible markers (eye colour, body colour, wing morphology), scoring could not be blinded to genotype; to limit observer bias, all measurements were taken from the recordings using the fixed criteria above rather than by live observation, and every recording could be re-inspected. Manual frame-based scoring of recorded negative geotaxis assays follows the logic of the photograph-based RING protocol [1]; fully automated alternatives exist [21,22] but were not used here.

For each individual fly, two pairs of variables were recorded:

- *Short_Distance_Height_cm*: the maximum vertical position (in cm) reached by the fly within the 10 s short-distance observation window, with 5.0 cm corresponding to the short-distance threshold. A value of 5.0 indicates the fly reached or exceeded the threshold, while lower values indicate the fly’s final position before timeout.
- *Short_Distance_Time_sec*: the elapsed time (in s) at which the fly’s position was recorded, equal to the latency to reach the 5 cm threshold for flies that succeeded, or the maximum observation time (10 s) for flies that did not reach the threshold.
- *Long_Distance_Height_cm* and *Long_Distance_Time_sec*: defined analogously for the 17.5 cm threshold within the 120 s observation window.

#### Data structure clarification

The raw dataset (S5 Table) contains 2,000 rows, one per fly measurement. The ‘Fly_Index’ column (values 1–10) numbers the 10 measurements recorded within each tube; it is a record number and does not encode climbing rank or order of measurement. Because the short-distance and long-distance values were scored as separate events, Fly_Index should not be interpreted as a persistent identity of one individual across the two assays. Each tube was tested only once. Accordingly, the 10 fly-level observations from a tube were aggregated into a single tube-level mean, and all subsequent statistical analyses were performed on these tube-level means (200 tubes total: 5 lines × 2 sexes × 20 tubes per line-sex combination). This aggregation ensures statistical independence between observations, avoids pseudoreplication arising from the spatial proximity of flies within the same tube, and makes the analysis independent of any assumption about individual identity within a tube.

### Data processing and statistical analysis

All analyses were performed using R software (version 4.3.0; R Core Team, 2023) with the dunn.test, rcompanion, and ggplot2 packages. The robustness analyses described below were run in R 4.3.3 using base R functions only; the complete script and its console output are provided as S1 File and S2 File.

#### Derived performance metrics

From the raw individual measurements, two complementary performance metrics were derived for each fly: climbing distance (cm), the height variable directly (Short_Distance_Height_cm, Long_Distance_Height_cm); and climbing speed (cm/s), calculated as Height ÷ Time for each fly, providing a continuous locomotor rate that is robust to ceiling effects when flies reach the threshold quickly. Climbing speed was adopted as the primary outcome variable because it incorporates elapsed time in addition to vertical position, avoiding the ceiling artefact observed for Oregon-R at the 5 cm threshold (see Results); threshold-based distance was retained as a secondary descriptive measure for direct comparison with prior studies [6,20].

#### Aggregation

For each replicate tube, the mean of the 10 individual flies was computed for each of the four metrics (short-distance height, long-distance height, short-distance speed, long-distance speed). All inferential tests were performed on these tube-level means (*n* = 20 tubes per line-sex cell; *n* = 40 per line pooled across sexes), satisfying the independence assumption.

#### Assumption checks

Normality within each line was assessed using the Shapiro-Wilk test, and homogeneity of variances across lines was evaluated using Levene’s test. Levene’s test indicated significant heterogeneity of variances for all four metrics (all *p* < 10^−5^), and the short-distance height of Oregon-R showed zero within-group variance due to a ceiling effect at the 5 cm cap. These violations precluded the use of classical parametric ANOVA, and non-parametric alternatives were therefore adopted as the primary inferential framework.

#### Inferential testing

The main effect of line was tested with the Kruskal-Wallis rank-sum test on tube-level means. When a significant main effect was detected, pairwise post-hoc comparisons between lines were performed using Dunn’s test with Bonferroni correction for multiple comparisons. The combined effect of line and sex (and their interaction) was assessed using the Scheirer-Ray-Hare test, the non-parametric two-way analogue of two-way ANOVA. Effect sizes for the Kruskal-Wallis tests are reported as epsilon-squared (ε^2^), where values of 0.01, 0.08, and 0.26 correspond to small, medium, and large effects, respectively. For adjacent lines in the performance hierarchy, Cliff’s delta was additionally computed as a distribution-free measure of separation between the two sets of tube-level means (δ = 1 indicates complete non-overlap), with 95% confidence intervals from 2,000 bootstrap resamples.

#### Robustness analyses

To assess how reproducible the line hierarchy is within the present dataset, four complementary resampling analyses were performed on the tube-level means (S6 Table; S1 File). (i) The male and female cohorts, which were reared, tested and scored independently (20 tubes per line each), were treated as two natural replicate experiments and their line rankings were compared. (ii) Split-half reliability: each line × sex cell of 20 tubes was randomly divided into two halves of 10 tubes, so that each half contained 20 tubes (200 flies) per line; the rank order of line means, the Kruskal-Wallis result and the significance of the four adjacent-pair Dunn comparisons were compared between halves over 2,000 random partitions. (iii) Bootstrap: tubes were resampled with replacement within line 5,000 times to obtain percentile 95% confidence intervals of line means and the proportion of resamples in which the full ranking Oregon-R > *w*^1118^ > *e*^1^ > *Ser*^1^ > *vg*^1^ was preserved. (iv) Small-cohort scenario: to mimic an independent replicate experiment of the size questioned during review (40 flies per line), 4 tubes per line (2 per sex; 40 flies) were drawn at random 5,000 times and the proportion of draws preserving the full ranking, and each adjacent pairwise ordering, was recorded. All resampling used a fixed random seed for reproducibility.

#### Reporting and significance

Results are expressed as mean ± standard error (SE) of tube-level means, with median and interquartile range (IQR) additionally reported in S1 Table given the use of rank-based tests. Statistical significance was accepted at *p* < 0.05. Complete output of all statistical tests is provided in Supplementary Tables S1-S4 and S6.

#### Visualization

All graphical visualizations were generated in R using the ggplot2 package. Box plots display tube-level distributions with median (central line), IQR (box limits), and 1.5×IQR whiskers; individual tube means are overlaid as jittered points. Significance markers above box plots are derived from Dunn’s post-hoc comparisons.

## Results

### General performance patterns

The climbing performance of *D. melanogaster* varied markedly among the tested lines across all four metrics examined: short-distance height (5 cm threshold), long-distance height (17.5 cm threshold), short-distance speed, and long-distance speed (Table 1; Figures 1 and 2). The Kruskal-Wallis test revealed a highly significant effect of line on tube-level means for every metric: short-distance height (H = 187.14, df = 4, *p* < 0.001, ε^2^ = 0.94), long-distance height (H = 190.36, df = 4, *p* < 0.001, ε^2^ = 0.96), short-distance speed (H = 190.29, df = 4, *p* < 0.001, ε^2^ = 0.96), and long-distance speed (H = 191.04, df = 4, *p* < 0.001, ε^2^ = 0.96). The epsilon-squared values exceed 0.9 across all metrics, indicating that line identity accounts for the overwhelming majority of variance in locomotor performance, while sex and tube-level random variation contribute only marginally.

**Figure 1.**
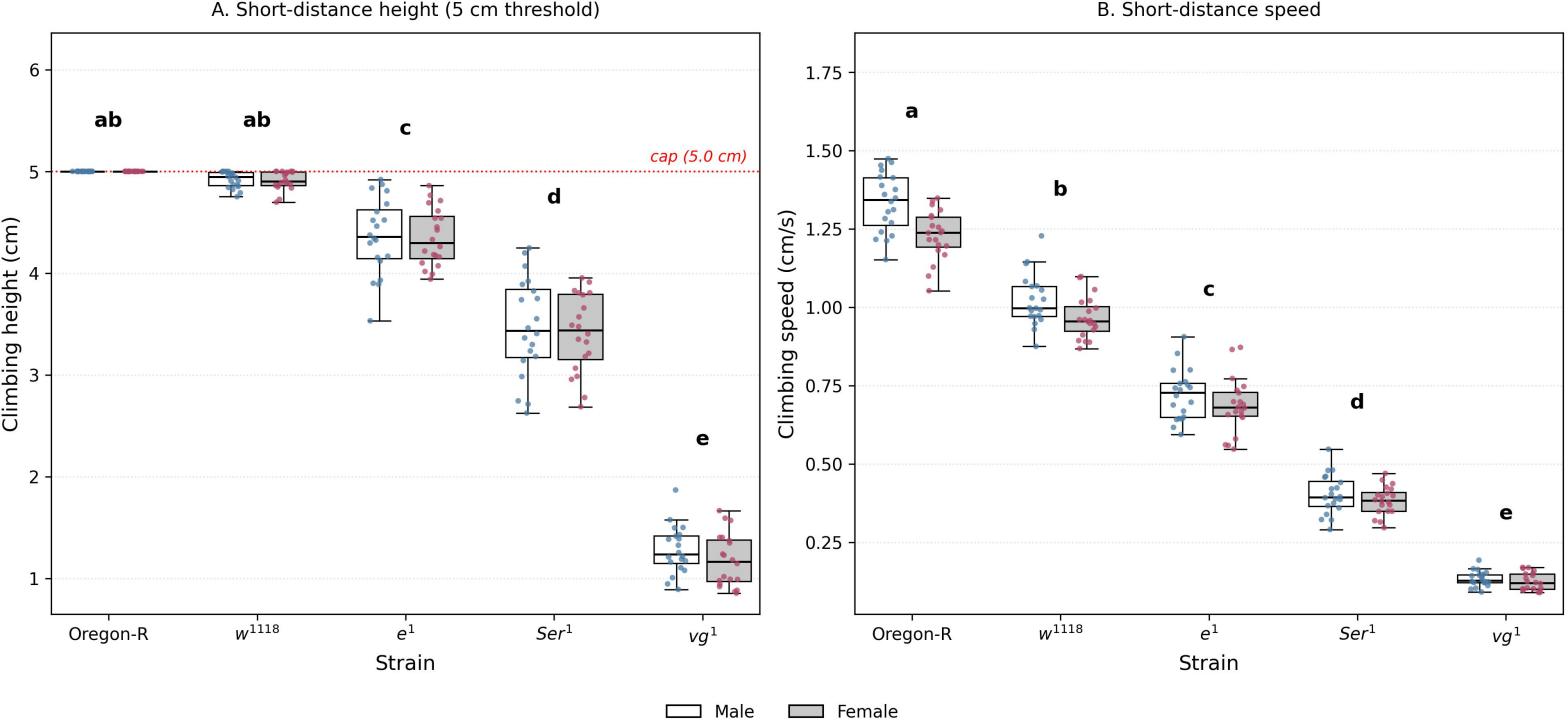
Short-distance climbing performance (5 cm threshold) across lines and sexes. (A) Climbing height (cm) per replicate tube. (B) Climbing speed (cm/s) per replicate tube. Box plots show data for males (white boxes) and females (grey boxes) across five *D. melanogaster* lines: Oregon-R (wild-type) *w*^1118^, *e*^1^, *Ser*^1^, and *vg*^1^. Each box displays the median (central line), interquartile range (IQR; box limits), and 1.5×IQR whiskers; individual tube means (*n* = 20 tubes per line × sex cell; each tube = mean of 10 individual flies) are overlaid as jittered points. The Kruskal-Wallis test revealed a highly significant effect of line on both metrics (height: H = 187.14, df = 4, *p* < 0.001; speed: H = 190.29, df = 4, *p* < 0.001). The Scheirer-Ray-Hare two-way test additionally confirmed no significant effect of sex (height: H = 0.04, df = 1, *p* = 0.84; speed: H = 0.72, df = 1, *p* = 0.40), and no significant line × sex interaction (both *p* > 0.99). Note the complete ceiling effect for Oregon-R in panel A (all 40 tubes reached the 5 cm cap, variance = 0); as a consequence, Dunn’s post-hoc could not distinguish Oregon-R from *w*^1118^ for the height metric (shared letters ‘ab’ in panel A; Bonferroni-adjusted *p* = 0.117). This is resolved in panel B, where the speed metric clearly separates all five lines (a, b, c, d, e). Letters above the boxes indicate Dunn’s post-hoc pairwise comparison groups corrected(Bonferroni-corrected, α = 0.05): lines sharing a letter do not differ significantly. Complete pairwise *p*-value matrices are provided in S3 Table.

**Figure 2.**
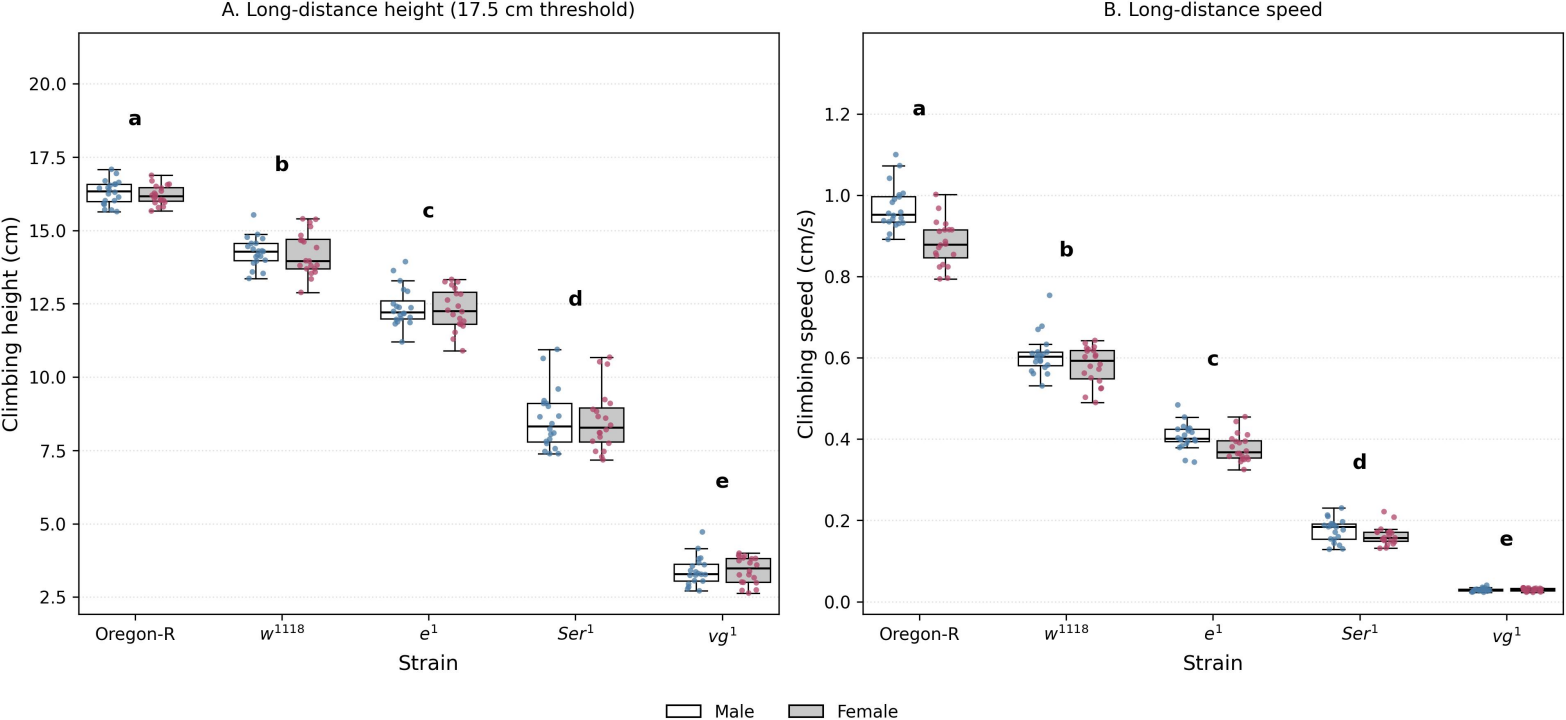
Long-distance climbing performance (17.5 cm threshold) across lines and sexes. (A) Climbing height (cm) per replicate tube. (B) Climbing speed (cm/s) per replicate tube. Box plots show data for males (white boxes) and females (grey boxes) across five *D. melanogaster* lines: type)Oregon-R (wild-type), *w*^1118^, *e*^1^, *Ser*^1^, and *vg*^1^. Each box displays the median (central line), interquartile range (IQR; box limits), and 1.5×IQR whiskers; individual tube means (*n* = 20 tubes per line × sex cell; each tube = mean of 10 individual flies) are overlaid as jittered points. The Kruskal-Wallis test revealed a highly significant effect of line on both metrics (height: H = 190.36, df = 4, *p* < 0.001; speed: H = 191.04, df = 4, *p* < 0.001). The Scheirer-Ray-Hare two-way test additionally confirmed no significant effect of sex (height: H = 0.02, df = 1, *p* = 0.90; speed: H = 0.64, df = 1, *p* = 0.42), and no significant line × sex interaction (both *p* > 0.97). The longer assay window (120 s) accentuates differences between lines relative to the short-distance assay, particularly for *Ser*^1^ and *vg*^1^ flies, supporting the use of the extended threshold for sensitive detection of motor deficits [6]. All five lines form distinct Dunn post-hoc groups in both panels (letters a, b, c, d, e from highest to lowest performance; Bonferroni-corrected, α = 0.05). Letters above the boxes indicate post-hoc comparison groups: lines sharing a letter do not differ significantly. Complete pairwise *p*-value matrices are provided in S4 Table.

**Figure 3.**
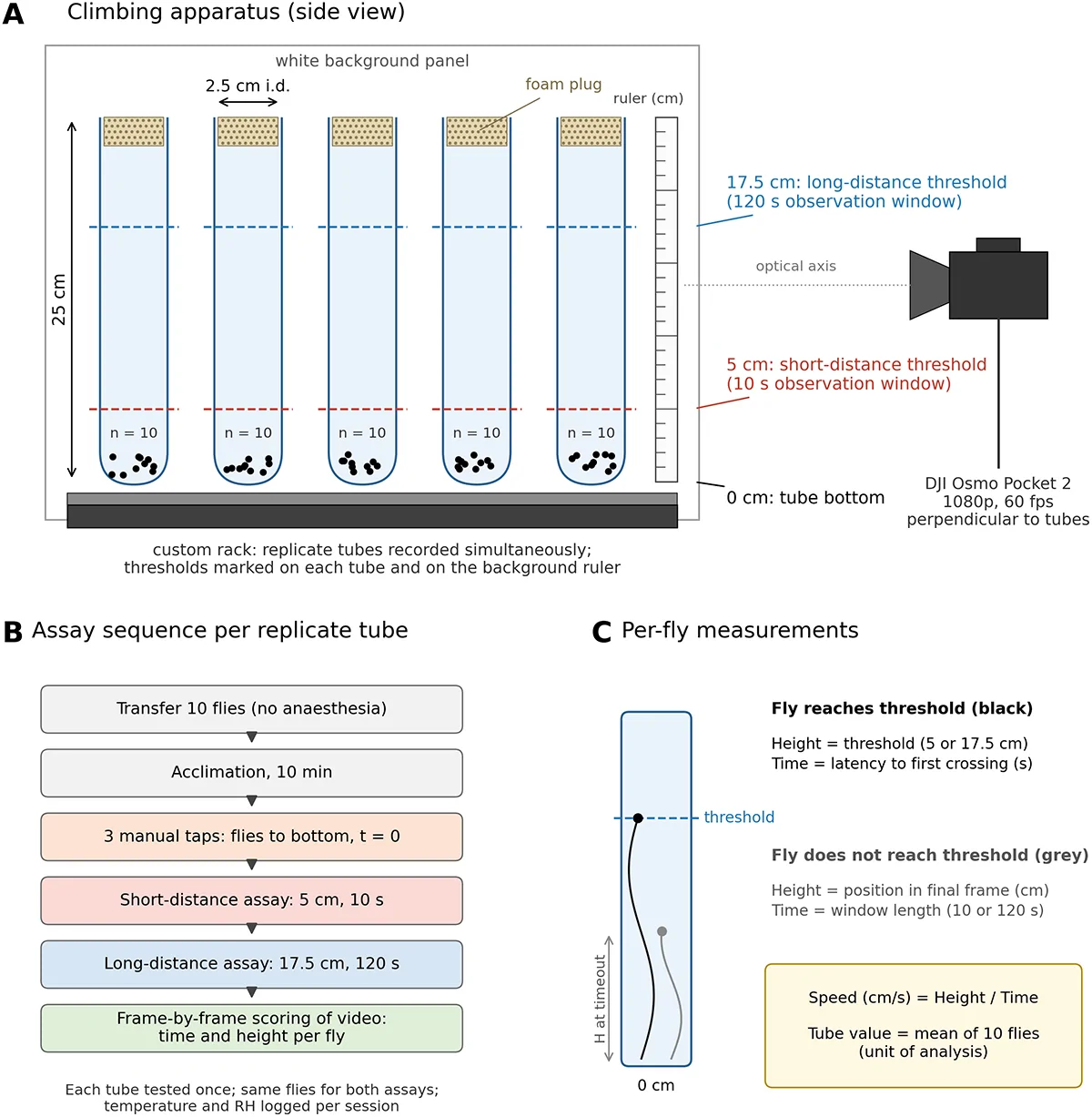
Schematic of the climbing apparatus, assay sequence and per-fly measurements. (A) Side view of the apparatus. Glass tubes (25 cm height, 2.5 cm internal diameter, rounded bottom, foam plug at the top) are mounted vertically on a custom rack in front of a white background panel carrying a centimetre ruler; the 5 cm (short-distance) and 17.5 cm (long-distance) thresholds are marked on each tube and on the ruler. Ten flies are placed in each tube. A DJI Osmo Pocket 2 camera (1920 × 1080 pixels, 60 frames s^−1^) on a tripod records all tubes in the rack simultaneously, perpendicular to the tubes. (B) Sequence of the assay for each replicate tube: transfer of 10 flies without anaesthesia, 10 min acclimation, three manual taps to bring the flies to the bottom (*t* = 0), short-distance assay (5 cm threshold, 10 s window), long-distance assay (17.5 cm threshold, 120 s window), and offline frame-by-frame scoring of the recording. Each tube was tested once; both assays used the same flies; temperature and relative humidity were logged per session. (C) Definition of the per-fly measurements. fly that reachesFor a fly that reaches the threshold (black trajectory), height equals the threshold and time is the latency to the first frame in which the threshold line is crossed. For a fly that does not reach the threshold within the window (grey trajectory), height is its vertical position in the final frame of the window, read against the ruler, and time is the window length. Climbing speed is height divided by time; the mean of the 10 flies gives the tube-level value used as the unit of analysis. The figure is a schematic representation, not to scale.

**Table 1.** Descriptive statistics for climbing performance across *Drosophila melanogaster* lines.

Line	Short Distance Height (cm)	Long Distance Height (cm)	Short Distance Speed (cm/s)	Long Distance Speed (cm/s)	*N*
Oregon-R	5.00 ± 0.00	16.25 ± 0.06	1.282 ± 0.016	0.926 ± 0.011	40
*w*^*1118*^	4.92 ± 0.01	14.25 ± 0.10	0.994 ± 0.013	0.594 ± 0.008	40
*e*^*1*^	4.36 ± 0.05	12.35 ± 0.11	0.703 ± 0.014	0.392 ± 0.006	40
*Ser*^*1*^	3.44 ± 0.07	8.54 ± 0.16	0.393 ± 0.009	0.169 ± 0.004	40
*vg*^*1*^	1.23 ± 0.04	3.40 ± 0.07	0.129 ± 0.004	0.029 ± 0.001	40

Notes: Values are mean ± standard error (SE) of tube-level means, pooled across both sexes. *N* = total number of replicate tubes per line (20 tubes per sex × 2 sexes); each tube contained 10 individual flies. Climbing speed was calculated for each individual fly as height ÷ time and then averaged within each tube. Short Distance threshold = 5 cm (10 s observation window); Long Distance threshold = 17.5 cm (120 s observation window). Lines: Oregon-R (wild-type), *w*^*1118*^ (*white*), *e*^*1*^ (*ebony*), *Ser*^*1*^ (*Serrate*), *vg*^*1*^ (*vestigial*). Kruskal-Wallis test for the main effect of line on tube-level means: short-distance height H = 187.14, long-distance height H = 190.36, short-distance speed H = 190.29, long-distance speed H = 191.04 (all df = 4, all *p* < 0.001, ε^2^ > 0.93). Median, IQR, and sex-stratified statistics are provided in S1 Table.

A consistent pattern emerged across both threshold-based and rate-based metrics: Oregon-R flies showed the highest performance, while *vg*^1^ flies exhibited the lowest, with intermediate values for *w*^1118^, *e*^1^, and *Ser*^1^. Detailed descriptive statistics (mean ± SE, median, and IQR) for each line-sex combination are provided in S1 Table; Kruskal-Wallis test statistics with effect sizes are summarized in S2 Table.

#### Ceiling effect at the 5 cm threshold

All Oregon-R flies reached the 5 cm short-distance threshold in every measurement, producing a within-group variance of zero for this metric and indicating a complete ceiling effect for the wild-type strain at this distance. As a consequence, Dunn’s post-hoc test could not statistically distinguish Oregon-R from *w*^1118^, the line with the next-highest performance (4.92 ± 0.01 cm; Bonferroni-adjusted *p* = 0.117, ns), for the short-distance height metric. The climbing speed metric (cm/s), which incorporates the time required to reach a given height, eliminated this ceiling and recovered the expected separation: Oregon-R short-distance speed (1.282 ± 0.016 cm/s) clearly differentiated the wild type from *w*^1118^ (0.994 ± 0.013 cm/s; Bonferroni-adjusted *p* = 0.027). For this reason, climbing speed serves as the primary outcome metric in subsequent comparisons, while threshold-based heights are retained as descriptive complements to enable comparison with previous studies [6,20].

### Sex-related comparison

The Scheirer-Ray-Hare test, the non-parametric two-way analogue of two-way ANOVA, revealed no significant effect of sex on any of the four metrics: short-distance height (H = 0.04, df = 1, *p* = 0.84), long-distance height (H = 0.02, df = 1, *p* = 0.90), short-distance speed (H = 0.72, df = 1, *p* = 0.40), and long-distance speed (H = 0.64, df = 1, *p* = 0.42). The line × sex interaction was likewise non-significant for all four metrics (all *p* > 0.97), indicating that the line-dependent performance hierarchy is consistent across both sexes.

When climbing ability was evaluated separately for each sex, the same line-dependent pattern persisted across all metrics (S1 Table). Oregon-R flies of both sexes exhibited the highest climbing performance, whereas *vg*^1^ flies consistently showed the lowest performance. Sex-specific comparisons for both distances are presented in Supplementary Figures S1 and S2. Together, these results indicate that locomotor performance under the standardized experimental conditions used in this study is determined by line identity rather than sex.

### Line-specific differences

Pairwise post-hoc comparisons using Dunn’s test with Bonferroni correction confirmed a pronounced line effect across both threshold and speed metrics. Complete pairwise matrices of adjusted *p*-values are presented in S3 Table (5 cm assay) and S4 Table (17.5 cm assay) for both height and speed.

Across all four metrics, the overall ranking of performance was consistent: Oregon-R > *w*^1118^ > *e*^1^ > *Ser*^1^ > *vg*^1^. Non-adjacent lines were separated with Bonferroni-adjusted *p*-values below 10^−5^ in every case, the strongest separations (e.g. Oregon-R vs *vg*^1^ across all four metrics) reaching *p* < 10^−30^. For lines adjacent in the hierarchy, the adjusted *p*-values were uniformly close to 0.02 (e.g. Oregon-R vs *w*^1118^: long-distance height *p* = 0.017, long-distance speed *p* = 0.020; *e*^1^ vs *Ser*^1^: short-distance height *p* = 0.028; all four adjacent contrasts for long-distance speed *p* = 0.020). These values should not be read as evidence of marginal or overlapping distributions. With 40 tubes per line and complete separation of two groups, the rank-based Dunn statistic reaches a fixed maximum (z = 3.09), which after Bonferroni correction over 10 comparisons yields exactly *p* = 0.020; the adjacent-pair values therefore represent the ceiling of the test rather than weak effects. This is confirmed by Cliff’s delta, which was 1.00 (complete non-overlap of tube-level distributions) for 11 of the 16 adjacent-pair contrasts and ≥0.93 for all others except Oregon-R vs *w*^1118^ for short-distance height (δ = 0.78), the one contrast affected by the ceiling at 5 cm (S6 Table). For that metric Oregon-R and *w*^1118^ did not differ significantly (*p* = 0.117), a direct consequence of the ceiling effect that collapsed both lines to the 5 cm cap; the climbing speed metric clearly resolved this distinction (*p* = 0.027), supporting its adoption as the primary outcome.

### Robustness of the line hierarchy

Because each line was tested in a single experiment, we asked how reproducible the hierarchy is within the dataset (S6 Table). First, the male and female cohorts, which were reared, tested and scored independently, produced identical rankings for all four metrics (Spearman ρ = 1.00; Pearson *r* ≥ 0.999 between the line means of the two cohorts). Second, in 2,000 random split-half partitions of the tubes, both halves reproduced the full ranking Oregon-R > *w*^1118^ > *e*^1^ > *Ser*^1^ > *vg*^1^, gave Kruskal-Wallis *p* < 0.001, and agreed on the significance of all four adjacent-pair Dunn comparisons in 100% of partitions for every metric. Third, in 5,000 bootstrap resamples of tubes within line, the percentile 95% confidence intervals of the line means did not overlap for any pair of lines on any metric, and the full ranking was preserved in 100% of resamples. Fourth, when independent cohorts of the size questioned during review were simulated by drawing only 4 tubes (40 flies) per line, the full ranking was recovered in 100% of 5,000 draws for all four metrics and the Kruskal-Wallis line effect was significant in every draw; the weakest single ordering, Oregon-R above *w*^1118^ for short-distance height, held in 99.9% of draws. Under the conditions of this study, therefore, an independent 40-fly cohort per line would be expected to reproduce the reported hierarchy with near certainty, and the effect sizes are large enough that the hierarchy is not sensitive to which subset of tubes is analysed.

As acknowledged in the limitations of this study, the lines used were not backcrossed to a common genetic background; therefore, the observed ranking reflects line-level differences that cannot be statistically attributed to the focal mutations alone, since uncontrolled background genetic variation is fully confounded with line identity in this design. The resampling analyses establish that the line differences are reproducible; they cannot establish their genetic cause. The magnitude of the differences observed, particularly between *vg*^1^ and the wild type, is nonetheless considerably larger than typically reported for standing background variation among laboratory *Drosophila* stocks [23,24], which is consistent with, but does not prove, a substantial contribution of the focal mutations to the locomotor phenotypes.

## Discussion

The line-dependent hierarchy observed here (Oregon-R > *w*^1118^ > *e*^1^ > *Ser*^1^ > *vg*^1^) was consistent across both distance- and speed-based metrics, across the two independently tested sexes, and across all resampling analyses. This convergence is consistent with a robust underlying difference in neuromuscular performance among the lines, although it should be interpreted with some caution: because climbing speed is mathematically derived from height and time, the two metrics are not fully independent, and their agreement does not by itself rule out that the line effect is being expressed through a single underlying dimension of variation. More importantly, the lines used in this study were not backcrossed to a common isogenic line prior to testing, and therefore the observed performance hierarchy may reflect residual background genetic variation in addition to, or instead of, the effects of the focal mutations. This issue is addressed in detail below.

### Genetic background and the choice of reference strain

Genetic background is a pervasive modifier of mutant phenotypes in *Drosophila*. Transcriptome differences between standard wild-type strains can be as large as those caused by mutations with substantial phenotypic effects [25]; background changes the expressivity of alleles, most strongly for alleles of intermediate effect, and alters complementation patterns even where the rank order of alleles is maintained [26]; and background-dependent severity can be conferred by autosomal and maternally inherited determinants, with a possible X-chromosomal contribution [27]. For the genes examined here the evidence is direct: multiple *vestigial* alleles show background-dependent expressivity, modulated by rearing temperature and sex [26], a major modifier of *scalloped* expressivity maps to the *vestigial* region [25], and in a classical backcrossing experiment the contribution of background, dominance and epistasis to geotactic behaviour exceeded the additive effect of markers at the *white* locus [28]. Startle-induced locomotion is likewise subject to pervasive epistasis that tends to suppress mutant effects in wild-derived backgrounds [29]. Because each of the four mutant lines used here carries its own historical background, line identity and focal allele are completely confounded in the present design, and the hierarchy we report is a property of the lines, not demonstrably of the alleles. This is why the study is framed, in its title and throughout, as a comparison of lines.

A related question is whether Oregon-R was an appropriate wild-type reference, given that many classical mutants were isolated or maintained in a Canton-S background and that Oregon-R and Canton-S differ in lifespan and other traits [30,31]. Three considerations apply. First, the two strains differ from each other in a trait-dependent manner rather than Oregon-R being uniformly more robust: Oregon-R lives longer than Canton-S and, most clearly in males, produces less mitochondrial ROS [31], Canton-S shows the lowest early mortality and the smallest ROS increase under hypoxia among Canton-S, Oregon-R and Berlin-K, with no difference in overall lifespan between Canton-S and Oregon-R in that study [32], locomotor senescence proceeds more rapidly in both Oregon-R and Canton-S than in the Samarkand and Lausanne-S wild-type strains, with no consistent advantage of one over the other [33], and, directly relevant to the present assay, Oregon-R flies show more pronounced rotenone-induced climbing deficits than Canton-S even though Canton-S survival is lower [34]. Under a high-throughput tracking paradigm, Canton-S, *w*^1118^ and Oregon-R also differ in movement explosiveness and endurance [35], and the performance of wild-type strains on exercise and climbing assays varies with background in ways reviewed by Damschroder et al. [36]. Second, adding Canton-S as a second reference would not remove the confound described above: nominally identical Canton-S sub-strains held in different laboratories differ markedly in locomotor behaviour [24], and the four mutant alleles used here have been maintained as independent stocks for decades, so none of them resides in a defined Canton-S background against which a Canton-S control would be informative. The appropriate control for each allele is a line into which the allele has been backcrossed, with periodic re-backcrossing to limit drift [27], which is the design we recommend for follow-up work. Third, Oregon-R is one of the two standard wild-type references in the negative geotaxis literature [1,3,33] and was the wild-type line used in our previous climbing study [18], which allows direct comparison of baseline values across our studies. Within these constraints, the present dataset should be read as a reproducible, fully specified baseline for five widely used lines under one protocol, and as a set of hypotheses about allele effects to be tested in background-controlled designs.

### Sex

The absence of a sex effect in this study deserves comment because the literature on sex differences in negative geotaxis is not uniform. A high-resolution automated assay revealed a previously unreported sexual dimorphism in the climbing behaviour of wild-type flies [14]; individual-trajectory analysis found females to climb better than males [37]; in DGRP lines males were naturally faster climbers than females, and mating status strongly affected female performance [19]; and in an automated RING assay the climbing ability of *w*^1118^ flies, males in particular, declined rapidly between days 14 and 21 [21]. In our earlier work with Oregon-R and *vestigial* flies, females showed a small advantage in climbing height (approximately 5%) already at day 5 that widened with age and was modulated by oxidative stress [18]. Two features of the present design are likely to have minimized sex differences: all flies were tested at a single young age (5 days), before the age-dependent divergence between the sexes documented in those studies [18,21], and females were tested as virgins, removing the reduction in climbing performance that follows mating [19]. A difference of the size seen at day 5 in our earlier study would, in any case, be small relative to the line effect here. The line effect was so large relative to any sex effect (ε^2^ > 0.93 for line versus H < 1 for sex) that a modest sex difference of the kind reported elsewhere would have been unlikely to alter the hierarchy in any case, as the identical rankings in the two sex cohorts show. Our conclusion is therefore restricted to young, virgin flies under standardized conditions, and should not be generalized to aged or mated animals.

### Interpretation of individual lines

The following interpretations relate the observed line phenotypes to the known functions of the mutated genes; given the background confound discussed above, they should be read as hypotheses consistent with the data rather than as demonstrated causal explanations.

The reduced performance of the *vg*^1^ line is consistent with the role of the *vestigial* gene in thoracic morphogenesis. Because *vestigial* mutations result in malformed thoracic musculature and defective balance organs [11–13], the impaired climbing ability plausibly reflects structural and neuromuscular constraints on motor coordination. At the molecular level, Vestigial acts as a transcriptional co-activator required for indirect flight muscle (IFM) differentiation, and loss of its function disrupts this programme, leading to defective muscle fibre differentiation and IFM degeneration [38]. If the climbing deficit of *vg*^1^ flies is attributable to the allele, it would reflect not only gross malformation of the thorax but also a failure in the transcriptional regulation of adult myogenesis. The size of the deficit (long-distance speed approximately 3% of the wild type) is well beyond the range of variation reported among wild-type backgrounds [23,24,36], which makes a substantial allelic contribution likely, but *vestigial* alleles are themselves known to show background-dependent expressivity [26], a modifier of wing phenotypes maps to the *vestigial* region [25], and the magnitude of the effect may differ in other backgrounds.

The intermediate but significantly reduced climbing performance of the *Ser*^1^ line can be interpreted in light of the developmental role of the *Serrate* gene in adult myogenesis. *Serrate* encodes a ligand of the Notch signalling pathway that drives proliferation of adult muscle precursor cells during larval development, generating the pool of myoblasts from which thoracic leg and flight muscles are subsequently formed [39,40]. Disruption of *Ser* function could therefore compromise this myoblast pool, potentially reducing the number and structural integrity of the muscle fibres that power locomotion. Notch signalling additionally regulates the specification of muscle attachment sites (myotendinous junctions) [41], whose integrity is critical for transmitting contractile force from muscle to exoskeleton. Taken together, the climbing deficit in *Ser*^1^ flies could reflect a convergence of reduced myoblast availability and impaired muscle-tendon connectivity arising from disrupted Notch-*Serrate* signalling during thoracic development [12,39,41].

In contrast, the *w*^1118^ and *e*^1^ lines demonstrated intermediate levels of performance, consistent with biochemical rather than structural defects. The *white* gene influences the transport of biogenic amines such as dopamine and serotonin, and its disruption can alter neurotransmission, leading to reduced behavioural responsiveness and visual deficits [9]. *white* has been directly implicated in motor function independently of its role in eye pigmentation: Xiao and Robertson [16] showed that *w* mutants exhibit delayed and more variable locomotor recovery from anoxia, and that pan-neuronal knockdown of *white* reproduces this phenotype while leaving eye pigmentation unaffected. Because dopaminergic and serotonergic signalling in the Mushroom Bodies are established regulators of *Drosophila* motor output [42,43], and because the *white* gene product is required for normal vesicular transport of both amines, its loss in *w*^1118^ flies would be expected to impair both motor-modulatory pathways, providing a candidate mechanism for the observed climbing deficit. Similarly, the *ebony* gene encodes a glial enzyme that conjugates biogenic amines; loss of *ebony* function reduces neurotransmitter levels and alters locomotor activity [10]. Because glial regulation of neurotransmitter homoeostasis is essential for normal motor circuit function [44], *e*^1^ flies may suffer from impaired glial neuromodulatory capacity that compromises motor circuits. The moderate but significant decline in climbing ability of these two lines is compatible with the notion that neurochemical imbalance can reduce muscular coordination even in the absence of gross anatomical deformities, although in both cases the contribution of the respective backgrounds remains to be separated experimentally.

### Broader relevance

Beyond their utility as classical genetic tools, the four mutant lines examined here map onto distinct classes of human neuromuscular and neurodegenerative pathology. The *vg*^1^ and *Ser*^1^ phenotypes, which are expected to arise from disrupted myogenesis and muscle-attachment formation, parallel structural myopathies and muscular dystrophies in which muscle fibre differentiation or the myotendinous junction is compromised. The *w*^1118^ and *e*^1^ phenotypes, which are expected to arise from impaired dopaminergic and serotonergic signalling, parallel neurotransmitter-deficit disorders such as Parkinson’s disease, where dopaminergic dysfunction produces a comparable progressive decline in motor output [45,46]. Because the climbing hierarchy observed here spans a gradient from lines with primarily neurochemical to lines with primarily structural deficits, this line panel may offer a tractable behavioural readout for future comparative work relating structural versus biochemical mechanisms of motor decline, provided that the alleles are first placed in a common background.

Together, these results highlight the reliability of the climbing assay as a behavioural biomarker of neuromuscular integrity in *Drosophila*, and provide a fully specified baseline (diet, apparatus, scoring procedure and raw data) against which future studies of these lines can be compared.

Overall, the present findings contribute to the growing understanding of how specific genetic alterations translate into behavioural phenotypes. Morphological mutations such as those in *vestigial* and *Serrate* are expected mainly to disrupt the physical machinery required for movement, whereas neurotransmitter-related mutations like *white* and *ebony* are expected to impair the signalling processes that coordinate muscular activity. The integration of these insights not only broadens the functional interpretation of classical *Drosophila* mutants but also provides a foundation for modelling human neuromuscular and neurodegenerative disorders, where both structural and neurochemical defects coexist [45,46].

### Limitations

Several limitations of this study should be acknowledged. First, and most importantly, the mutant lines were not backcrossed to a common genetic background, so allele and background are confounded, and the performance hierarchy is attributable to lines rather than to the focal mutations (see above). Regular backcrossing to a standard background is recommended for non-lethal mutants precisely to avoid this problem [27], and a definitive test of the allelic hypotheses generated here requires co-isogenic lines, multiple alleles per gene, RNAi-mediated knockdown, or transgenic rescue. Second, each line was tested in a single experiment. The resampling analyses (S6 Table) show that the hierarchy is fully reproducible within the dataset, including in simulated 40-fly cohorts, and the two sex cohorts constitute independently reared and scored replicates; however, they cannot substitute for an independent repetition in a different season, with different food batches, or in another laboratory, and variation in performance among wild-type strains [36] and between laboratories holding nominally the same strain [24] is well documented. Third, only one wild-type reference (Oregon-R) was used, as it is the only wild-type strain held in our collection; the reasons why a Canton-S reference would not have resolved the background issue are given above, but because the rank order of wild-type strains in climbing assays is itself variable [36], a panel of several wild-type strains would give a better estimate of the range of normal variation against which mutant deficits should be judged. Fourth, scoring was performed manually by a single observer who could not be blinded to genotype because the mutant phenotypes are externally visible; although all measurements were taken from recordings using fixed criteria, automated tracking [21,22] would remove this potential source of bias in future work. Fifth, the 5 cm short-distance threshold produced a complete ceiling effect for Oregon-R (variance = 0; all flies reached the cap in every measurement). Although the parallel climbing speed metric resolved this issue analytically, future studies adopting only threshold-based outcomes should consider higher cut-offs or alternative continuous measures to avoid loss of resolution at the upper end of the performance distribution. Finally, all flies were tested at a single age and females as virgins; the absence of a sex effect should not be extrapolated beyond these conditions.

## Conclusions

This study demonstrates that locomotor performance in *D. melanogaster* is largely shaped by line identity rather than sex. The wild-type Oregon-R strain exhibited the strongest climbing ability, whereas the lines carrying morphological (*vg*^1^, *Ser*^1^) and neurotransmitter-related (*w*^1118^, *e*^1^) mutations showed progressively reduced performance. The hierarchy was identical in both sexes, in every split-half partition and in every bootstrap resample of the data, and would be expected to reproduce in an independent 40-fly cohort per line under the same conditions. These results are consistent with, but because of the uncontrolled genetic backgrounds do not demonstrate, the expectation that both structural and biochemical disruptions impair neuromuscular coordination. The consistency of the performance hierarchy across climbing distances and across both threshold- and rate-based metrics confirms the robustness of the negative geotaxis assay as a behavioural measure of motor integrity. Future research employing isogenic backgrounds, RNAi knockdown, or transgenic rescue is needed to clarify the precise contribution of each gene. Taken together, our findings provide a reproducible, fully documented baseline for five widely used *Drosophila* lines and underscore their value as complementary models for exploring the genetic and physiological bases of movement disorders and neuromuscular decline.

## Supplementary Material

Supplemental Material

## Data Availability

The data underlying the findings reported in this manuscript are available as Supplementary Material. Raw individual climbing measurements for all 2,000 flies across all line-sex combinations and experimental conditions are provided in S5 Table. Summary statistics, test statistics, effect sizes, and post-hoc comparisons are provided in S1-S4 Tables; robustness analyses are provided in S6 Table. All analyses reported in this manuscript can be reproduced from S5 Table using the R packages dunn.test, rcompanion, and ggplot2; the robustness analyses can be reproduced with the base R script provided as S1 File, whose console output is given in S2 File.
